# MeCP2 confers 5-fluorouracil resistance in gastric cancer via upregulating the NOX4/PKM2 pathway

**DOI:** 10.1186/s12935-022-02489-y

**Published:** 2022-02-18

**Authors:** Yannan Qin, Xiaoping Ma, Chen Guo, Shuang Cai, Hailin Ma, Lingyu Zhao

**Affiliations:** 1 Department of Cell Biology and Genetics/Key Laboratory of Environment and Genes Related To Diseases, School of Basic Medical Sciences, Xi’an Jiaotong University Health Science Center, Xi’an, 710061 Shaanxi China; 2 Institute of Genetics and Developmental Biology, Translational Medicine Institute, School of Basic Medical Sciences, Xi’an Jiaotong University Health Science Center, Xi’an, 710061 Shaanxi China; 3 Department of Radiation Oncology, The First Affiliated Hospital of Medical College, Xi’an Jiaotong University, Xi’an, 710061 Shaanxi China

**Keywords:** MeCP2, Gastric cancer, 5-Fluorouracil resistance, NOX4

## Abstract

**Background:**

Increasing evidence suggests that aberrant methylation is involved in 5-fluorouracil (5-FU) resistance in gastric cancer (GC). Our previous work has identified that Methyl-CpG binding protein 2 (MeCP2) promotes GC progression by binding to the methylation sites of promoter regions of specific genes to affect the downstream signaling pathways. However, the function and molecular mechanisms of MeCP2 in GC 5-FU resistance remain unclear.

**Methods:**

We detected the expression of MeCP2 in 5-FU-resistant GC cells and examined cell behaviors when MeCP2 was silenced. The molecular mechanisms were explored through chromatin immunoprecipitation (ChIP)-qRT-PCR, luciferase reporter assay, clinical tissue samples analysis, and in vivo tumorigenicity assay.

**Results:**

MeCP2 was up-regulated in 5-FU-resistant GC cells. Knockdown of MeCP2 enhanced the sensitivity of the cells to 5-FU. Moreover, MeCP2 promoted NOX4 transcription in the cells by binding to the promoter of NOX4. Silencing NOX4 rescued the inductive effect of MeCP2 overexpression on 5-FU sensitivity of GC cells and reduced the expression of NOX4 and PKM2 in MeCP2 overexpressed 5-FU-resistant GC cells. In addition, our in vivo experiments demonstrated that MeCP2 knockdown enhanced 5-FU sensitivity in tumors.

**Conclusion:**

MeCP2 confers 5-FU resistance in GC cells via upregulating the NOX4/PKM2 pathway, which may lead to a promising therapeutic strategy for GC.

**Supplementary Information:**

The online version contains supplementary material available at 10.1186/s12935-022-02489-y.

## Background

Gastric cancer (GC) remains the fourth most common malignancy and the third main cause of malignancy-related death worldwide [1, 2]. China is the country with the highest GC occurrence, with an estimated number of 370,000 new cases each year, taking up over 40% of the global annual cancer incidence [3]. The standard treatment for GC is surgical resection of operable tumors in combination with local radiotherapy or chemotherapy with conventional anticancer drugs [4]. Currently, chemotherapies based on 5-FU are widely used in clinical treatment for cancers [5]. In GC, the application of 5-FU is limited because the drug results in frequent chemoresistance. The inherent or acquired resistance of GC patients to 5-FU therapy is a major clinical issue, while the mechanisms underlying the development of 5-FU chemoresistance in patients remain scarcely understood.

DNA methylation is a frequent epigenetic event that plays some important role in cancer development. Cancer cells may become drug resistant and escape apoptosis through DNA methylation and other epigenetic modifications. For example, GC patients with p16INK4a methylation were reported to specifically benefit from 5-FU adjuvant chemotherapy [6], and methylation of PYCARD and DAPK1, the apoptosis-related genes, resulted in resistance to 5-FU and poor prognoses in GC patients [7]. Gene expression profiles and DNA methylation levels in drug-resistant GC cells revealed that 74 genes were hypermethylated and downregulated (e.g., ATP2C2), or hypomethylated and upregulated (e.g., ARC) in 5-FU-resistant cells [8]. In addition, an increasing number of genes with aberrant methylation, including ASCL2, PCDH17, DCTPP1, TFAP2E, DACT2 and TFAP2E, are found to be related to 5-FU resistance in GC [9–11].

Methyl-CpG binding protein 2 (MeCP2), a methylated binding protein, regulates chromatin organization and gene transcription by binding to methylated DNA or gene promoters. MeCP2 has been identified to be a key oncogene in cancer development [12]. Our previous works have found that MeCP2 promotes HEPG2 cell proliferation in human hepatocellular carcinoma by activating ERK1/2 and inhibiting p38 activity [13] and facilitates breast cancer growth by promoting ubiquitination-mediated P53 degradation by binding to the RPL5/RPL11 promoter regions to suppress RPL5/RPL11 transcription [14]. We have also found that MeCP2 promotes GC cell proliferation through FOXF1-mediated Wnt5a/β-Catenin signaling pathway and suppresses cell apoptosis through MYOD1-mediated Caspase-3 signaling pathway [15], or facilitates GC cell proliferation through activation of the MEK1/2-ERK1/2 signaling pathway by upregulating GIT1 [16], and it regulates glycogenes directly or indirectly to alter glycopatterning and affect GC cell proliferation and apoptosis [17]. Our most recent research reveals that MeCP2 inhibits miR-22, resulting in deficiency of endogenous S-adenosylmethionine, which finally leads to tumor suppressor dysregulation [2]. These studies demonstrate that MeCP2 promotes GC progression by binding to the methylated sites on promoter regions of genes to active or inhibit the downstream signaling pathways. However, it remains unknown whether MeCP2 contributes to 5-FU resistance in GC, and the potential function and molecular mechanism of that are unclear. In this study, we examined the MeCP2 expression in 5-FU-resistant GC cells and cell behaviors in MeCP2-silenced 5-FU-resistant GC cells. Chromatin immunoprecipitation (ChIP)-qRT-PCR and luciferase reporter assay were performed to examine the downstream genes regulated by MeCP2. Our results confirmed that MeCP2 knockdown improved the 5-FU sensitivity of 5-FU-resistant GC cells, and the effect of MeCP2 was exerted via regulating the NOX4/PKM2 pathway.

## Methods

### Cell lines and 5-FU

Human GC cell lines BGC-823 and SGC-7901 as well as normal gastric mucosa cell line GES were obtained from the Cell Bank (Shanghai Genechem Co., Ltd., Shanghai, China). All the cell lines have been tested and authenticated by the corporation. 5-FU-resistant cells, BGC823/5FU and SGC7901/5FU, were obtained from Cell Bank of Chinese Academy of Sciences (Shanghai, China). The cells were maintained in RPMI-1640 medium (Gibco-BRL, NY, USA) supplemented with 10% fetal bovine serum (Gibco, NY, USA) at 37 °C and 5% CO_2_. The anticancer agent 5-FU was purchased from CSNpharm (Chicago, USA).

### siRNA synthesis, plasmid construction, and transfection

siRNAs targeting MeCP2 and NADPH oxidase 4 (NOX4) were designed and synthesized by GenePharma Corporation (SGC, Shanghai, China). The negative control was a scrambled sequence siRNA (NC-siRNA). The siRNA sequences are listed in Additional file 1: Table S1. Full-length human MeCP2 complementary DNA was cloned into pCMV2-GV146 vector. pCMV2-GV146-GFP-MeCP2 plasmid (WT), pCMV2-GV146-GFP-Mutation type 1 plasmid (MT1) and pCMV2-GV146-GFP-Mutation type 2 plasmid (MT2) were constructed (Additional file 1: Table S2) [15]. MT1 and MT2 are mutants with defects at two different sites in the MBD domain of MeCP2 so that their proteins cannot bind to the methylated CpG sites of target genes. The reporter plasmid pGL3-NOX4, which contained a 220-bp fragment spanning from 89,323,211 to 89,323,430 located at the promoter region of NOX4, was placed at the downstream of the Firefly Luciferase reporter gene (pGL3-NOX4-luc) (Genechem Co. Ltd., Shanghai, China). BGC-823 and SGC-7901 cells were seeded for 24 h in RPMI-1640 medium without antibiotics, following which siRNAs and/or plasmid vectors targeting specific genes were transiently transfected into cells by using Lipofectamine 2000 (Invitrogen, Carlsbad, CA, USA) and cells were cultured for 48 h for subsequent examinations.

### RNA extraction and qRT-PCR

Total RNA was extracted from the cell lines and frozen tissues using TRIzol Reagent (Invitrogen, Carlsbad, CA, USA) following the manufacturer's instructions. FFPE tissue samples (10 sections) were deparaffinized by incubation for 10 min in xylene and 5 min in 100% ethanol, and were washed with distilled water for 30 s, followed by RNA extraction using the Qiagen FFPE Rneasy Kit (Valencia, CA, USA). The RNA samples were examined spectrophotometrically using Nanodrop (Thermo Fisher Scientific Inc., DE, USA). cDNA was synthesized following the manufacturer's protocol (Takara, Dalian, China). qRT-PCR was conducted using the SYBR Green PCR kit (Takara Biotechnology, Dalian, China). The primers are presented in Additional file 1: Table S3. The qRT-PCR reactions were performed in triplicate for each sample using the IQ5 Multicolor qRT-PCR Detection System (Bio-Rad, USA). β-Actin was used as mRNA control. The 2^−ΔΔCt^ method was adopted for the analysis.

### Protein extraction and Western blotting

Total protein was extracted from the tissue samples and GC cells using RIPA buffer (Cell Signaling Technology, Boston, MA) supplemented with protease inhibitors (Roche, Indianapolis, IN, USA). Protein was quantified using the BCA Protein Assay Kit (Pierce Biotechnology, Rockford, USA). Equal amounts of protein lysates were run on 10% SDS-PAGE gels and electro blotted onto polyvinylidene difluoride (PVDF) membranes. The membranes were then blocked with 5% non-fat milk overnight and incubated for 2 h the next day with a 1:1000 dilution of anti-MeCP2 antibody, anti-NOX4 antibody (Santa Cruz Biotechnology), or anti-PKM2 antibody (Abcam). After washing with PBS three times and incubation with appropriate secondary antibody for 1 h, protein visualization was performed by using enhanced chemiluminescence (Pierce Biotechnology, Rockford, USA).

### MTT assay for cell viability

Briefly, BGC-823, SGC-7901, BGC-823/5-FU and SGC-7901/5-FU cells were seeded in 96-well plates (5 × 10^2^ cells/well) respectively and cultured at 37 °C for 24 h. The cells were then treated with 5-FU at the concentration of 0, 0.1, 0.5, 1, 5, 10, 20, 40, 80 or 160 μM for 48 h, after which MTT (2.0 mg/ml) was added to each well and the plates were incubated in darkness at 37 °C for 2 h. Subsequently, the medium was removed, formazan crystals were dissolved in DMSO, and optical density (OD) was measured using an ELISA plate reader at 570 nm. The cell viability index was calculated according to the formula: (experimental OD value/control OD value) × 100%.

### Colony formation assay

Cells were planted in 6-well plates (1 × 10^3^ cells/well), treated with 10 μM 5-FU for 48 h, transfected with specific si-RNA and/or overexpression plasmid, and incubated in a humidified atmosphere with 5% CO_2_ at 37 °C for a week. The cell colonies were then fixed with 4% paraformaldehyde for 15 min at room temperature and stained with crystal violet for another 15 min. After washing with PBS, the colonies were recorded by Syngene GBox (Syngene, Cambridge, UK) and only clearly visible colonies with a diameter over 50 μm were counted.

### Cell cycle assay

Cells were harvested for analysis by trypsinization 48 h after transfection, washed twice with PBS and fixed with ice-cold ethanol (70%) at 4 °C overnight. After washing twice again, the cells were incubated with Rnase A (0.1 mg/ml) and propidiumiodide (PI, 0.05 mg/ml) for 15 min at room temperature. Then, distribution of cell-cycle stages was detected by flow cytometry (FACS Calibur, BD Biosciences, CA, USA).

### Apoptosis analysis

Cells were harvested 48 h after transfection, washed twice with PBS and stained using the Annexin V-FITC/PI Apoptosis Detection kit (Invitrogen, Carlsbad, CA, USA). Flow cytometry was performed and the stained cells were counted to quantify cell apoptosis.

### Chromatin immunoprecipitation (ChIP)-qRT-PCR

BGC-823 cells were transfected with empty plasmid, WT, MT1 or MT2. ChIP was conducted as previously described [15]. Briefly, BGC-823 cells were cross-linked with 1% formaldehyde and quenching was performed using 125 mM glycine. The chromatin was sonicated into 200-bp (approx.) fragments. Cell lysates were divided into two portions and incubated respectively with 5 μg antibody against IgG or against MeCP2 or GFP (Abcam, Cambridge, MA, USA, Additional file 1: Table S5) overnight at 4 °C. DNA–protein complexes were captured and eluted in TE buffer. After decrosslinking, DNA was extracted using the QIA quick PCR purification kit (QIAGEN, Germany) and analyzed by qRT-PCR using gene-specific primers (Additional file 1: Table S4).

### Luciferase reporter assay

BGC-823 cells were seeded into 96-well culture plates (4 wells per group). pGL3-NOX4-luc was amplified in DH5α and was treated with CpG methyltransferase M. Sssl (M0226S, NEB, USA) for 48 h (pGL3-NOX4-luc + Methylation). The cells were transfected with pGL3-luc, pGL3-NOX4-luc, or pGL3-NOX4-luc + Methylation plasmids for 48 h, after which cells (except those transfected with pGL3-luc) were treated with NC-siRNA, MeCP2 siRNA-1/2, null vector, and ov-mecp2 vector, respectively, for 48 h. Then, the luciferase activity per 1000 cells (trypan blue staining) was measured.

### Tissue specimens

Tissue samples of GC and adjacent normal gastric mucosa (5–10 cm away from the primary tumors) of the same patient were collected from 81 patients receiving surgical management at the First Affiliated Hospital of Xi’an Jiaotong University (Xi’an, China) from March, 2017 to January, 2020. The tissues were snapfrozen in liquid nitrogen and stored at − 80 °C. The tissue sections from each patient were reviewed by two experienced pathologists. All patients had not received radiotherapy or chemotherapy before surgery. Follow-up was performed post surgery. The study was conducted following the protocol approved by the ethics committee of the university, and written informed consent was obtained from all patients.

### Tumorigenicity assay in nude mice

A total of 12 male, 5-week-old BALB/C nude mice were used to examine tumorigenicity. The animal maintenance and experimental procedures were approved by the Institutional Animal Care and Use Committee of Xi'an Jiaotong University. The mice were divided into three groups (n = 4): sh-Ctrl + PBS, sh-MeCP2 + PBS and sh-MeCP2 + 5-FU. In brief, group one was transplanted with BGC-823/5-FU cells stably expressing sh-Ctrl, while groups two and three were transplanted with cells expressing sh-MeCP2 (1 × 10^6^). Then, 5-Fu at 60 mg/kg of body weight or PBS was injected intraperitoneally one time a week for a total of 5 weeks. At the 35th day, the nude mice were anesthetized through inhalation of 3% isofluorane and given one subcutaneous dose of carprofen (8 mg/kg) for euthanasia. Then xenograft tumors were excised, weighed and pictured. Tumor volume (V) was examined by measuring the tumor length (L) and width (W) and calculated according to the formula: V = (L × W^2^)/2. The tumor tissues were frozen for qRT-PCR and Western blotting, and embedded in paraffin for immunohistochemistry.

### Immunohistochemisty

The FFPE tissue samples, including GC patient specimens and xenograft tumor tissues from the nude mice, were sectioned at 4-μm thickness. The sections were deparaffinized with xylene and hydrated with graded alcohol for antibody staining. Then the sections were incubated with primary antibody against MeCP2 (Santa Cruz, CA, USA) at a dilution of 1:200, followed by incubation with secondary antibody. Then, 3,3ʹ-diaminobenzidine (DAB) and hematoxylin were performed. MeCP2 expression was considered high when the proportion of positive cells was over 50% in 5 random fields.

### Statistical analysis

All analyses were performed in the GraphPad Prism program. Data were presented as the mean ± standard error of the mean (SEM) with statistical significance indicated when P < 0.05. Student’s t-test or one-way variance analysis was used for inter-group comparisons.

## Results

### MeCP2 was up-regulated in 5-FU-resistant GC cells

**Fig. 1:**
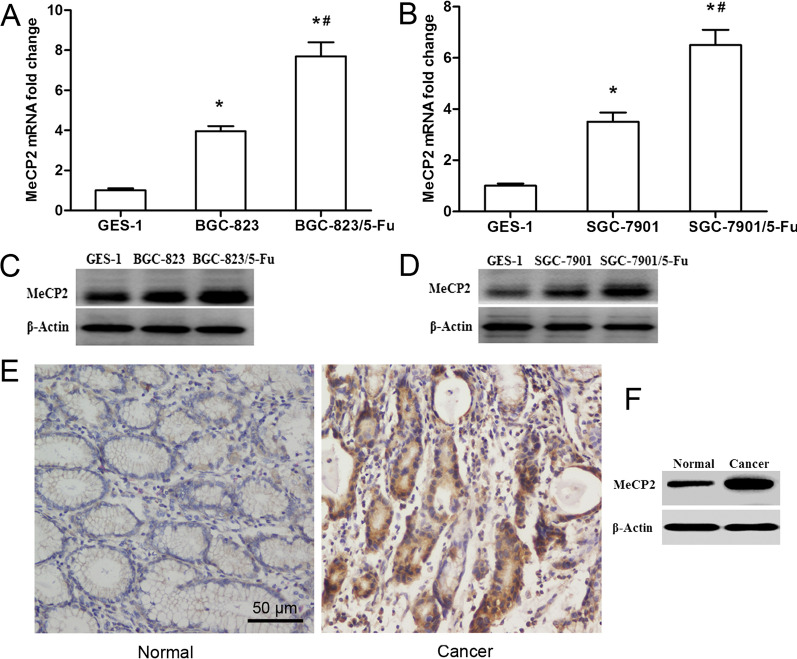
MeCP2 expression in 5-FU-resistant GC cells. The mRNA (**A**, **B**) and protein (**C**, **D**) levels of MeCP2 in 5-FU-resistant GC cells (BGC-823/5-FU and SGC-7901/5-FU) vs. in regular GC cells (BGC-823 and SGC-7901). The expression of MeCP2 in GC and normal tissues by immunohistochemistry (**E**) and WB test (**F**)

To capture the potential difference in MeCP2 expression between GC cells and 5-FU-resistant GC cells, qRT-PCR and western blot were conducted to measure the level of MeCP2 in BGC-823, SGC-7901, BGC-823/5-FU and SGC-7901/5-FU cells, respectively. As demonstrated by the results, the mRNA (Fig. 1A, B) and protein (Fig. 1C, D) expressions of MeCP2 were noticeably up-regulated in 5-FU-resistant GC cells (BGC-823/5-FU and SGC-7901/5-FU) as compared with in GC cells (BGC-823 and SGC-7901). Besides, the expression of MeCP2 protein was significantly higher in GC tissues than in normal gastric tissues (Fig. 1E, F) [15].

### MeCP2 knockdown improved the 5-FU sensitivity of 5-FU-resistant GC cells

**Fig. 2:**
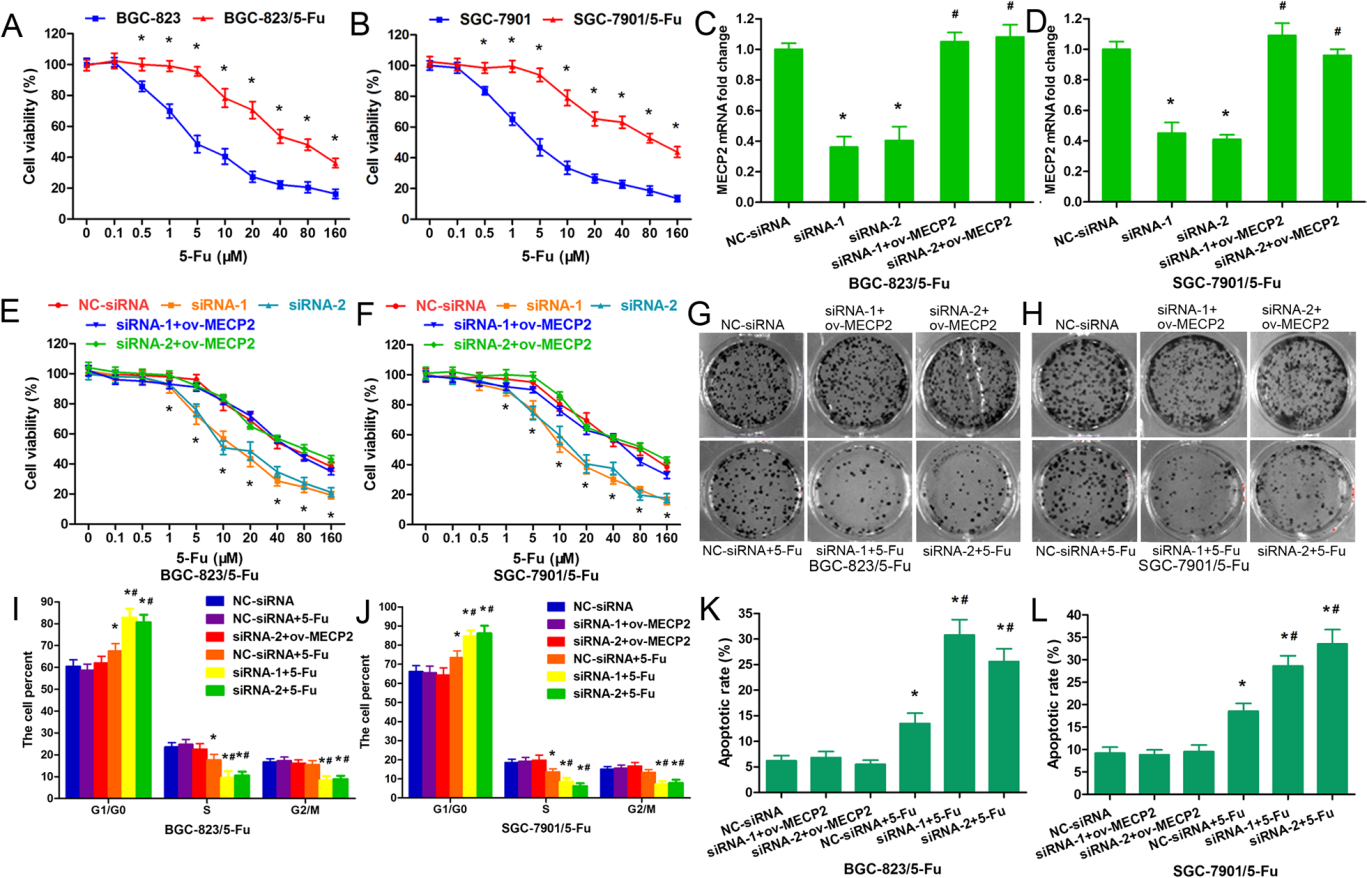
MeCP2 knockdown improved the 5-FU sensitivity of 5-FU-resistant GC cells. Cell viability in GC cells and 5-FU-resistant GC cells treated respectively with 0, 0.1, 0.5, 1, 5, 10, 20, 40, 80 and 160 μM 5-FU for 48 h (**A**, **B**). Interferential efficiency of MeCP2 in 5-FU resistant GC cells transfected with siRNA-1 or siRNA-2 targeting to MeCP2, or NC-siRNA, or siRNA-1 or siRNA-2 plus MeCP2-overexpressing plasmid (*p < 0.05, as compared with NC-siRNA; ^#^p < 0.05, as compared with siRNA-1 or siRNA-2; Student's t-test) (**C**, **D**). Cell viability (**E**, **F**), colony formation (**G**, **H**), cell cycle (**I**, **J**) and cell apoptosis (**K**, **L**) were detected in 5-FU resistant GC cells transfected with siRNA-1 or siRNA-2 targeting to MeCP2, or with NC-siRNA, or with siRNA-1 or siRNA-2 + ov-MeCP2, and then treated with 5-FU (*p < 0.001, as compared with NC-siRNA; ^#^p < 0.001, as compared with NC-siRNA + 5-FU; Student's t-test)

Cell viability was determined by MTT assay in GC cells and 5-FU-resistant GC cells treated with various concentrations of 5-FU. It was found that 5-FU exerted a growth inhibitory effect on cells in a dose-dependent manner (Fig. 2A, B). However, such effect was not as obvious in 5-FU-resistant GC cells as in regular GC cells (Fig. 2A, B).

To further explore the role of MeCP2 in 5-FU resistant GC cells, BGC-823/5-FU and SGC-7901/5-FU cells were transfected with siRNA-1 or siRNA-2 targeting MeCP2, or NC-siRNA, or siRNA-1 or siRNA-2 plus MeCP2-overexpressing plasmid (ov-MECP2) and then treated with 10 μM 5-FU. qRT-PCR analysis revealed that MeCP2 was remarkably reduced in cells transfected with either siRNA-1 or siRNA-2, but increased remarkably again in cells transfected with siRNA-1 or siRNA-2 + ov-MECP2 (Fig. 2C, D). Knockdown of MeCP2 enhanced the cell sensitivity to 5-FU but there is no significant change in siRNA-1 or siRNA-2+ ov-MECP2 transfected cells (Fig. 2E, F). To further explore the effect of MeCP2 on 5-FU related cell proliferation, examination of colony formation, cell cycle and apoptosis were performed. As indicated by the results, MeCP2 silencing induced a more distinctive decrease of cell colony in BGC-823/5-FU and SGC-7901/5-FU cells than in control cells (Fig. 2G, H). Meanwhile, MeCP2 siRNA led to a greater increase in G1 phase cells (Fig. 2I), a greater decrease in S phase cells (Fig. 2J), and a greater increase of early and late apoptotic cells (Fig. 2K, L) in the 5-FU resistant cells (Fig. 2I, J). Taken together, down-regulation of MeCP2 could overcome the issue of GC cell resistance to 5-FU.

**Fig. 3:**
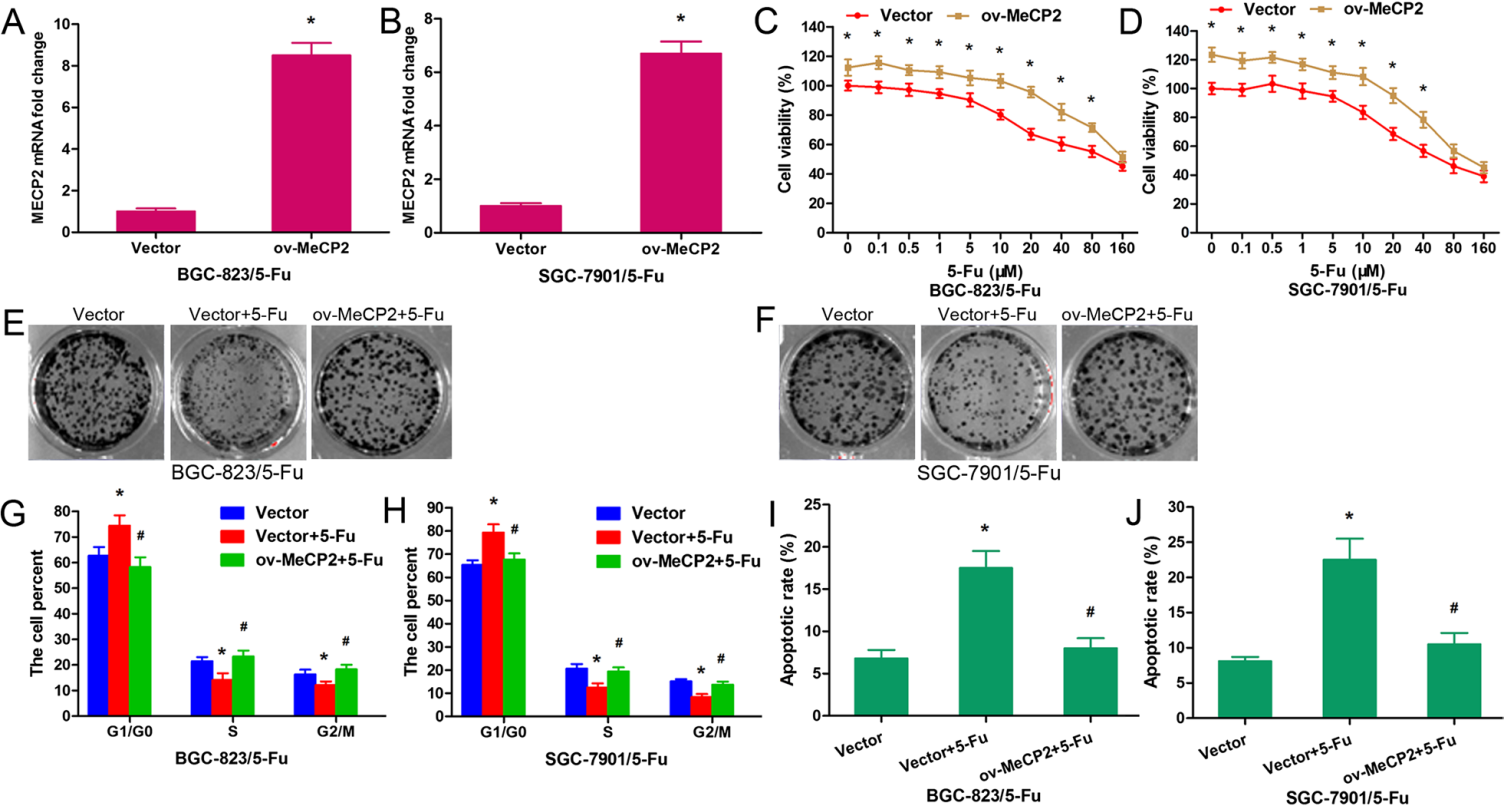
MeCP2 overexpression inhibited the 5-FU sensitivity of 5-FU-resistant GC cells. **A**, **B** Overexpressing efficiency of MeCP2 in 5-FU resistant GC cells transfected with MeCP2-overexpressing plasmid or empty plasmid. Cell viability (**C**, **D**), colony formation (**E**, **F**), cell cycle (**G**, **H**) and cell apoptosis (**I**, **J**) were detected in 5-FU resistant GC cells transfected with MeCP2-overexpressing plasmid or empty plasmid followed by 5-FU treatment (*p < 0.001, as compared with empty plasmid; ^#^p < 0.001, as compared with empty plasmid + 5-FU; Student's t-test)

We further constructed a MeCP2-overexpressing plasmid. The plasmid efficiently up-regulated the MeCP2 levels in BGC-823/5-FU and SGC-7901/5-FU cells (Fig. 3A, B). Based on cell viability and colony formation analyses, up-regulation of MeCP2 reduced the sensitivity of BGC-823/5-FU and SGC-7901/5-FU cells to 5-FU (Fig. 3C–F). Cell cycle analysis showed that MeCP2 overexpression reversed the alteration of cell cycles in BGC-823/5-FU and SGC-7901/5-FU cells (Fig. 3G, H). Meanwhile, MeCP2 overexpression significantly reduced early and late apoptotic cells, which also reversed the effect of apoptosis in BGC-823/5-FU and SGC-7901/5-FU cells (Fig. 3I, J).

### MeCP2 promoted NOX4 transcription by binding to NOX4 promoter

**Fig. 4:**
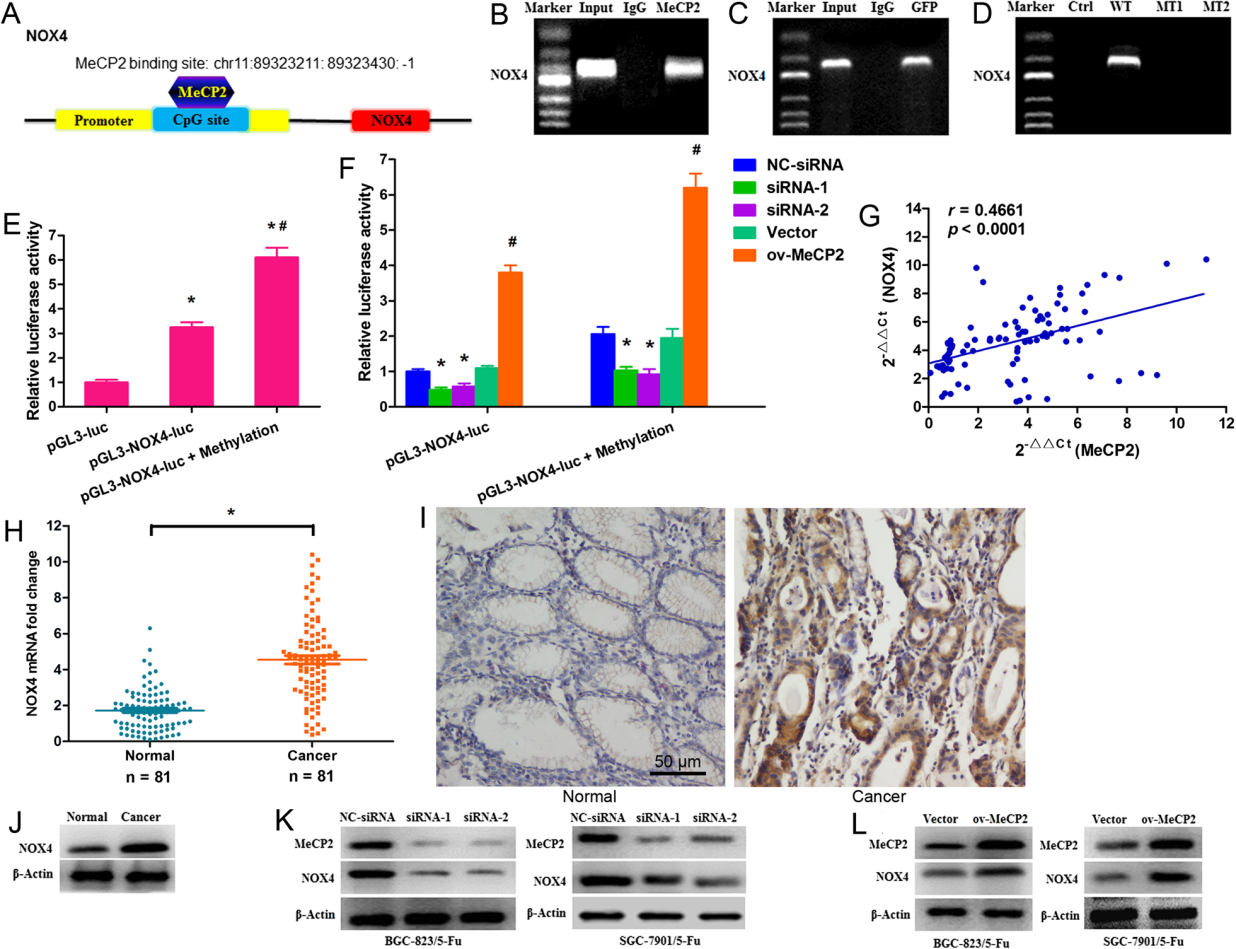
MeCP2 promoted NOX4 transcription by binding to its promoter in 5-FU-resistant GC cells. **A** The MeCP2 binding site on the promoter region of NOX4. **B–D** ChIP RT-PCR of NOX4 was performed respectively with anti-MeCP2 antibody, anti-GFP antibody after transfection with GFP-MeCP2 plasmid, and anti-GFP antibody after transfection with Ctrl (GFP plasmid), WT (GFP-MeCP2 plasmid), MT1 (GFP- Mutation type 1) or MT2 (Mutation type 2). **E** BGC-823/5-FU cells were transfected with pGL3-luc, pGL3-NOX4-luc (target sequence of NOX4 promoter region), or pGL3-NOX4-luc + Methylation. Luciferase activity was determined at 48 h post-transfection. Renilla luciferase served as the internal control. Data are shown as mean ± SEM (*p < 0.001, as compared with pGL3-luc; ^#^p < 0.001, as compared with pGL3-NOX4-luc; Student's t-test). **F** BGC-823/5-FU cells were transfected with NC-siRNA, MeCP2 siRNAs, MeCP2 overexpression plasmid, or null vector, and treated with pGL3-NOX4-luc or pGL3-NOX4-luc + Methylation, respectively. Luciferase activity was determined at 48 h post-transfection. Data are shown as mean ± SEM (*p < 0.001, as compared with NC-siRNA; ^#^p < 0.001, as compared with null vector; Student's t-test). **G** MeCP2 and NOX4 levels were positively correlated in 81 pairs of GC tissue and normal para-carcinoma tissue (PCT). The 2^−ΔΔCt^ values of MeCP2 and NOX4 mRNA were subjected to a Pearson correlation analysis (r = 0.466, n = 81, p < 0.0001, Pearson's correlation). **H** NOX4 mRNA expression in GC tissues and PCTs. Data are shown as mean ± SEM (*p < 0.01, Student's t-test). **I** NOX4 protein expression in GC tissues versus normal PCTs by immunohistochemistry. **J** NOX4 protein expression in GC tissues versus normal PCTs (pooled sample). **K** Silencing MeCP2 down-regulated the expression of NOX4 in 5-FU-resistant BGC-823 and SGC-7901 cells. **L** Overexpressed MeCP2 promoted the expression of NOX4 in the cells

NOX4 is the most frequently overexpressed isoform of NOX in cancer cells [18]. It is localized at the inner mitochondria membrane, functions as a mitochondrial energetic sensor coupling cancer metabolic reprogramming to drug resistance, and mediates drug resistance through PKM2 [19]. In our previous study, chr11:89,323,211:89,323,430 (negative strand) at the promoter region of NOX4 (Fig. 4A) was captured by the antibody against MeCP2 (10 folds enrichment) in BGC-823 cells [15]. In this study, we used the GFP-MeCP2 plasmids, including wild type (WT) and mutation type (MT1 and MT2), constructed by us previously [15]. The sequence chr11:89,323,211:89,323,430 was predicted to have the CpG site and the primers of the sequence were synthesized. After the plasmid was transfected into BGC-823/5-FU cells, ChIP RT-PCR was conducted using anti-MeCP2 antibody or anti-GFP antibody. The results suggested that exogenous MeCP2 bound to the sequence in the cells (Fig. 4B, C). GFP plasmid (Ctrl), MT1 and MT2 did not bind to the sequence, but WT did (Fig. 4D). Then, a promoter reporter assay was conducted to determine whether MeCP2 bound to the sequence. It was found that the luciferase activity significantly increased in the pGL3-NOX4-luc and pGL3-NOX4-luc + Methylation groups as compared with in the pGL3 group, and the activity was higher in the pGL3-NOX4-luc + Methylation group than in the pGL3-NOX4-luc group (Fig. 4E). When the pGL3-NOX4-luc plasmid was transfected into the cells, the luciferase activity decreased in the MeCP2 siRNA-1 and siRNA-2 groups as compared with in the NC-siRNA group, and increased in the MeCP2 overexpression group as against the empty vector group. Transfection with the pGL3-NOX4-luc + Methylation plasmid induced similar results, but the luciferase activity in the group was higher than in the pGL3-NOX4-luc group (Fig. 4F). To further reveal the relationship between MeCP2 and NOX4, we examined their mRNA expressions in 81 paired tissue samples using qRT-PCR. A significant positive correlation between MeCP2 mRNA and NOX4 mRNA was identified (Fig. 4G). Both mRNA and protein levels of NOX4 were significantly higher in GC tissues than in normal gastric tissues (Fig. 4H–J). Patient characteristics and clinicopathologic correlation of NOX4 expression was listed in Additional file 1: Table S6. In addition, the NOX4 protein level decreased in BGC-823/5-FU and SGC-7901/5-FU cells after transfection with MeCP2 siRNA-1 or siRNA-2 (Fig. 4K), and increased in the cells after transfection with the MeCP2 overexpression vector (Fig. 4L). In short, our results suggested that MeCP2 promoted the expression of NOX4 in GC by binding to the specific sequence chr11:89,323,211:89,323,430 at its promoter region.

### *MeCP2 conferred 5-FU resistance *via* upregulating the NOX4/PKM2 pathway*

**Fig. 5:**
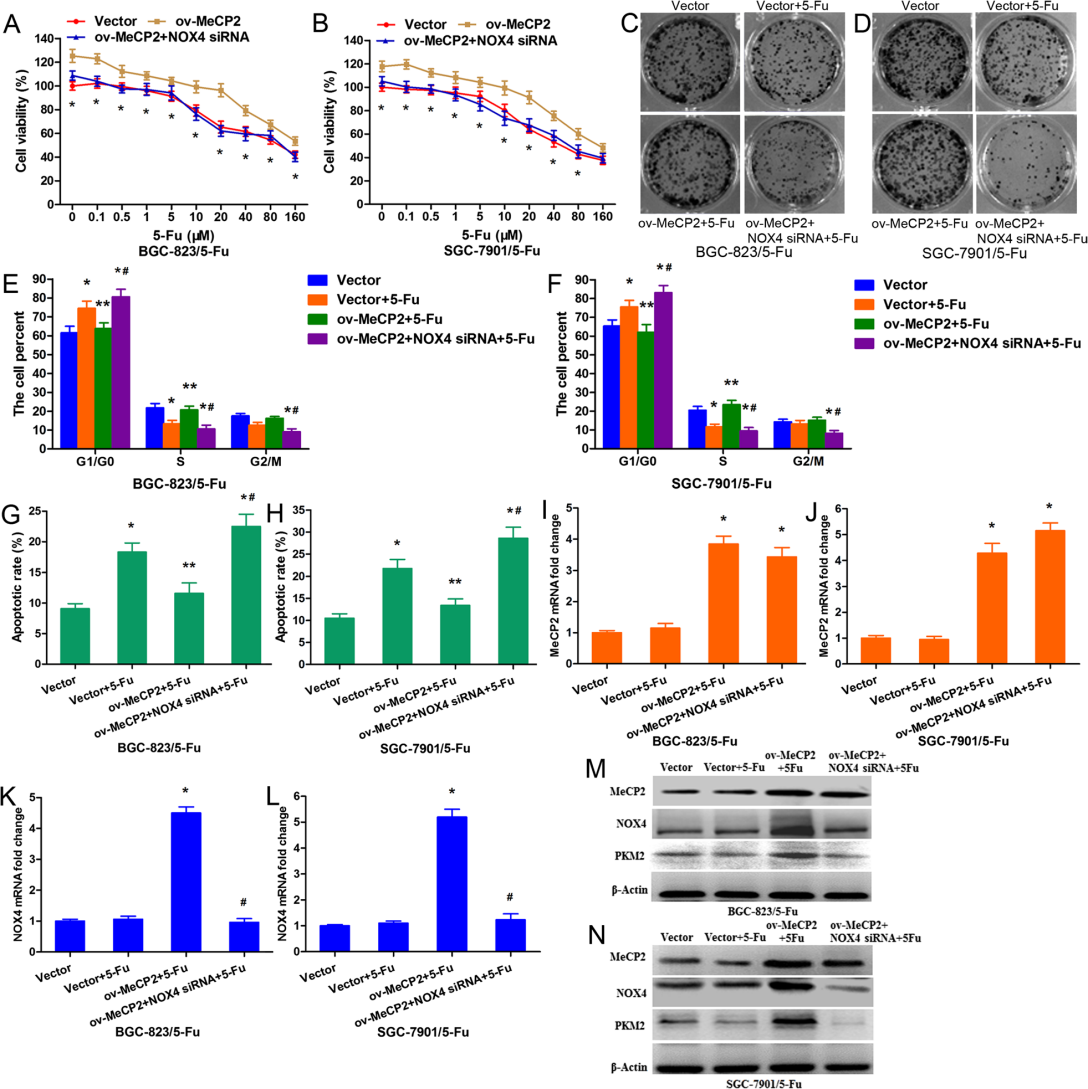
MeCP2 confers 5-FU resistance via upregulating the NOX4/PKM2 pathway. 5-FU-resistant BGC-823 and SGC-7901 cells were co-transfection with MeCP2 overexpressing plasmid and NOX4 siRNA. **A**, **B** MTT assay for cell viability (*p < 0.01). **C**, **D** Cell colony assay 12 days after co-transfection. **E**, **F** Percentages of cells in the G1/G0, S, and G2/M phases after co-transfection. **G**, **H** Percentages of early-apoptotic and late-apoptotic cells after cotransfection. Data are shown as mean ± SEM (*p < 0.01, as compared with empty plasmid; **p < 0.01, as compared with empty plasmid + 5-FU; ^#^p < 0.01, as compared with MeCP2 overexpressing plasmid + 5-FU; Student's t-test, n = 3). **I–L** mRNA levels of MeCP2 and NOX4 after cotransfection (*p < 0.01, as compared with empty plasmid; ^#^p < 0.01, as compared with MeCP2 overexpressing plasmid + 5-FU). **M**, **N** Protein levels of MeCP2, NOX4 and PKM2 after cotransfection

To further confirm that MeCP2 contributed to 5-FU resistance by promoting NOX4 expression, NOX4 siRNA was synthesized and was co-transfected with MeCP2-overexpressing plasmid into BGC-823/5-FU and SGC-7901/5-FU cells treated with 5-FU. As revealed by our results, overexpression of MeCP2 resulted in promoted cell proliferation, while silencing of NOX4 rescued such effect (Fig. 5A–D). MeCP2 overexpression induced a significant decrease of cells at the G1 phase and a concomitantly remarkable increase of cells at the S phase in both BGC-823/5-FU and SGC-7901/5-FU cells (Fig. 5E, F), and it also induced a notable decrease in both early and late apoptotic cells (Fig. 5G, H), Whereas, such effects were reversed by the co-transfection with MeCP2-overexpressing plasmid and NOX4-siRNA. We further performed qRT-PCR and Western blot analyses to determine the expressions of MeCP2 and NOX4, as well as the downstream PKM2. It was found that the mRNA expression of MeCP2 was significantly up-regulated in both BGC-823/5-FU and SGC-7901/5-FU cells transfected with MeCP2-overexpressing plasmid alone or in combination with NOX4 siRNA (Fig. 5I, J); whereas, the mRNA level of NOX4 significantly increased in both cells after transfection with MeCP2-overexpressing plasmid but decreased after cotransfection with MeCP2-overexpressing plasmid plus NOX4 siRNA (Fig. 5K, L). The protein expressions of MeCP2 and NOX4 showed the same trend. Similar to NOX4, PKM2 was up regulated in BGC-823/5-FU and SGC-7901/5-FU cells transfected with MeCP2-overexpressing plasmid alone but decreased in cells co-transfected with MeCP2-overexpressing plasmid plus NOX4 siRNA (Fig. 5M, N).

### MeCP2 knockdown enhanced 5-FU sensitivity in tumors in vivo

**Fig. 6:**
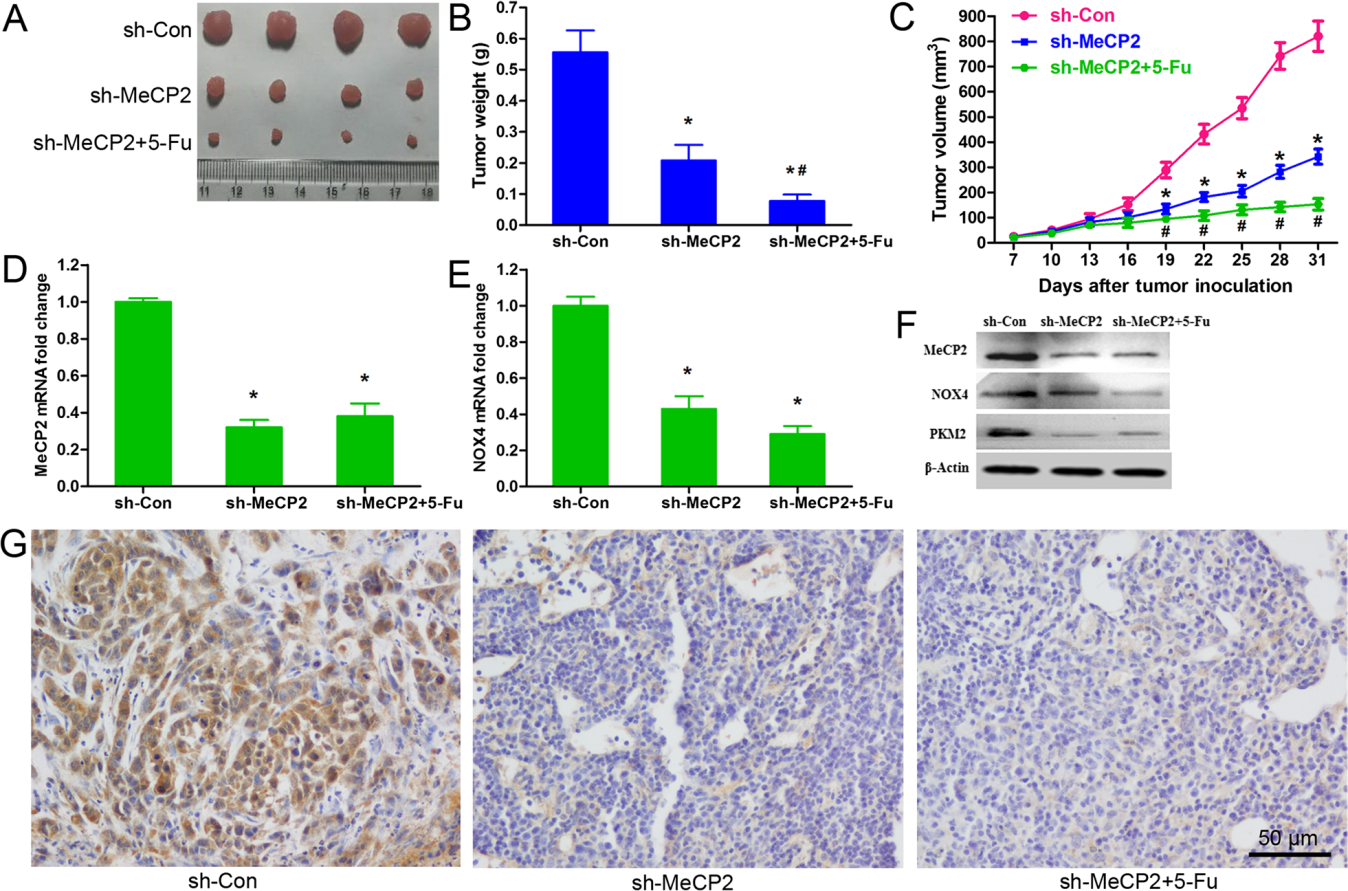
MeCP2 knockdown enhanced 5-FU sensitivity in tumors in vivo. Xenograft tumors excised from mice inoculated with BGC823/5-FU cells stably expressing sh-MeCP2 or sh-con and treated with 5-FU or PBS. **A** Tumor images. **B**, **C** Tumor weight and volume (*p < 0.01, as compared with empty plasmid; ^#^p < 0.01, as compared with MeCP2 overexpressing plasmid). **D**, **E** qRT-PCR for the mRNA levels of MeCP2 and NOX4 (*P < 0.05). **F** Western blot for the protein levels of MeCP2, NOX4 and PKM2. **G**. IHC staining of MeCP2

The effect of MeCP2 silencing on the cytotoxic effect of 5-FU was further validated in vivo using a mouse GC xenograft model. Compared with that in the sh-con + PBS group, the tumor in the sh-MeCP2 + PBS group was lighter and smaller, and these effects were reinforced by 5-FU treatment (Fig. 6A–C). In addition, knockdown of MeCP2 downregulated the expressions of MeCP2 and NOX4 mRNAs regardless of 5-FU treatment (Fig. 6D, E). In line with this, Western blot analysis revealed lower protein levels of MeCP2, NOX4 and PKM2 in the sh-MeCP2 + PBS and sh-MeCP2 + 5-FU groups (Fig. 6F). Immunohistochemistry demonstrated that the protein expression of MeCP2 was obviously downregulated in both of sh-MeCP2 + PBS and sh-MeCP2 + 5-FU groups (Fig. 6G).

## Discussion

Chemoresistance has severely restricted the therapeutic outcome in GC patients. To identify the underlying mechanism of chemoresistance and develop therapeutic strategies against it is of great importance. The present study reveals that MeCP2 expression is remarkably up regulated in 5-FU-resistant GC cells and that knockdown of MeCP2 could improve the sensitivity of the cells to 5-FU. More importantly, MeCP2 contributes to the GC cell resistance to 5-FU by binding to the the specific sequence chr11:89,323,211:89,323,430 at the promoter region of NOX4 to promote its transcription and activate the NOX4/PKM2 pathway. Collectively, MeCP2 works as a positive regulator in the resistance to 5-FU in GC, and targeting MeCP2 may be an effective scheme to deal with GC chemoresistance.

Previous studies have identified changes in DNA methylation status of tumor suppressor genes and oncogenes that could be used as biomarkers for diagnosis and prognosis in cancer patients undergoing chemotherapies. MeCP2, an important member of the methyl-CpG-binding domain family, has been found overexpressed in primary GC tissues and involved in regulating cell proliferation and apoptosis [2, 13–17]. It is reported to be related to multidrug resistance gene 1 (MDR1) [20] and be highly expressed in camptothecin-resistant cells [21]. In addition, MeCP2 and miR-19a/b are found to repress reciprocally to regulate multidrug resistance in GC cells [22]. To evaluate the effectiveness and function of MeCP2 in 5-FU resistance in GC, this study examined the expression of MeCP2 and cell behaviors in MeCP2 silenced 5-FU resistant GC cells, as well as the tumorigenicity in nude mice. Our results showed that MeCP2 expression was higher in 5-FU-resistant GC cells than in regular GC cells, and silencing MeCP2 inhibited cell proliferation, blocked the cell cycle to G1-S phase, and promoted cell apoptosis of 5-FU-resistant GC cells. The in vivo examination found that the tumor was lighter and smaller when MeCP2 was silenced, and such impact was reinforced by the treatment of 5-FU. All these findings confirm that MeCP2 confers 5-FU resistance in GC cells. Targeting MeCP2 may be a potentially effective way to overcome the resistance to 5-FU in GC patients and hence to improve the prognosis of these patients.

The present study further explored the functional mechanism of how up-regulated MeCP2 contributes to 5-FU resistance in GC. In our previous work [15], the sequence chr11:89,323,211:89,323,430 (negative strand) at the promoter region of NOX4 was captured by the anti-MeCP2 antibody in BGC-823 cells. In this study, our ChIP-qRT-PCR and luciferase reporter assays confirm that MeCP2 could bind to chr11:89,323,211:89,323,430. As is reported in some studies, MeCP2 in neurological diseases acts as both a transcriptional repressor by selectively binding to methylated CpG dinucleotides and recruiting co-repressors such as histone deacetylases and Sin3A, and a transcriptional activator by selectively binding to methylated CpG islands and recruiting activators such as CREB110 [23]. In our study, exogenous MeCP2 promoted NOX4 transcription in BGC-823/5-FU cells, meanwhile NOX4 was significantly highly expressed in GC tissues. NOX4 is one of the seven NOX family members that transport electrons from NADPH to oxygen, generating hydrogen peroxide (H_2_O_2_) and ROS superoxide anion (O_2_^−^) [24]. It has been found to be involved in several cancer cell lines in regulating cell proliferation [25], invasion [26], and migration [27], as well as epithelial-mesenchymal transition (EMT) and invadopodia formation [28]. In addition, a previous study reported that silencing NOX4 through PKM2 sensitized cultured and ex vivo freshly isolated human-renal carcinoma cells to drug-induced cell death [19], and another study reported that EFHD2 promoted cisplatin resistance in non-small cell lung cancer by activating the NOX4-ROS-ABCC1 axis [18]. In the present study, silencing NOX4 mitigated the effect of MeCP2 overexpression on cell proliferation, cell cycle and cell apoptosis. The expressions of NOX4 and PKM2 significantly increased in MeCP2-overexpressing cells but decreased when NOX4 was silenced. These results demonstrate that MeCP2 confers 5-fluorouracil resistance in GC via upregulating the NOX4/PKM2 pathway.

## Conclusions

In summary, the present study demonstrates that MeCP2 knockdown improves the sensitivity of GC cells to 5-FU treatment by regulating the transcription of NOX4 to affect the NOX4/PKM2 pathway. This indicates a promising therapeutic target to overcome 5-FU resistance in GC.

## Supplementary Information

**Additional file 1.** Sequences, antibodies, and patient characteristics in this study.

## Data Availability

The datasets used and/or analyzed during the current study are available from the corresponding author on reasonable request.

## References

[1] Xie L, Cai L, Wang F, Zhang L, Wang Q, Guo X (2020). Systematic review of prognostic gene signature in gastric cancer patients. Front Bioeng Biotechnol.

[2] Tong D, Zhang J, Wang X, Li Q, Liu L, Lu A, Guo B, Yang J, Ni L, Qin H, Zhao L, Huang C (2020). MiR-22, regulated by MeCP2, suppresses gastric cancer cell proliferation by inducing a deficiency in endogenous S-adenosylmethionine. Oncogenesis.

[3] Guo F, Kong WN, Feng YC, Lv J, Zhao G, Wu HL, Ai L, Zhou X, Cai XL, Sun W, Ma XM (2020). Comprehensive analysis of the expression and prognosis for MCMs in human gastric cancer. Technol Cancer Res Treat.

[4] Liu YP, Ling Y, Qi QF (2013). The effects of ERCC1 expression levels on the chemo sensitivity of gastric cancer cells to platinum agents and survival in gastric cancer patients treated with oxaliplatin-based adjuvant chemotherapy. Oncol Lett.

[5] Nakamura A, Nakajima G, Okuyama R, Kuramochi H, Kondoh Y, Kanemura T, Takechi T, Yamamoto M, Hayashi K (2014). Enhancement of 5-fluorouracil-induced cytotoxicity by leucovorin in 5-fluorouracil-resistant gastric cancer cells with upregulated expression of thymidylate synthase. Gastric Cancer.

[6] Mitsuno M, Kitajima Y, Ide T, Ohtaka K, Tanaka M, Satoh S (2007). Aberrant methylation of p16 predicts candidates for 5-fluorouracil-based adjuvant therapy in gastric cancer patients. J Gastroenterol.

[7] Kato K, Iida S, Uetake H, Takagi Y, Yamashita T, Inokuchi M (2008). Methylated TMS1 and DAPK genes predict prognosis and response to chemotherapy in gastric cancer. Int J Cancer.

[8] Maeda O, Ando T, Ohmiya N, Ishiguro K, Watanabe O, Miyahara R (2014). Alteration of gene expression and DNA methylation in drug-resistant gastric cancer. Oncol Rep.

[9] Kwon OH, Park JL, Baek SJ, Noh SM, Song KS, Kim SY (2013). Aberrant upregulation of ASCL2 by promoter demethylation promotes the growth and resistance to 5- fluorouracil of gastric cancer cells. Cancer Sci.

[10] Zhang Y, Fan J, Fan Y, Li L, He X, Xiang Q, Mu J, Zhou D, Sun X, Yang Y, Ren G, Tao Q, Xiang T (2018). The new 6q27 tumor suppressor DACT2, frequently silenced by CpG methylation, sensitizes nasopharyngeal cancer cells to paclitaxel and 5-FU toxicity via β-catenin/Cdc25c signaling and G2/M arrest. Clin Epigenetics.

[11] Jingyue S, Xiao W, Juanmin Z, Wei L, Daoming L, Hong X (2019). TFAP2E methylation promotes 5-fluorouracil resistance via exosomal miR-106a-5p and miR-421 in gastric cancer MGC-803 cells. Mol Med Rep.

[12] Müller HM, Fiegl H, Goebel G, Hubalek MM, Widschwendter A, Müller-Holzner E, Marth C, Widschwendter M (2003). MeCP2 and MBD2 expression in human neoplastic and non-neoplastic breast tissue and its association with oestrogen receptor status. Br J Cancer.

[13] Zhao LY, Zhang J, Guo B, Yang J, Han J, Zhao XG, Wang XF, Liu LY, Li ZF, Song TS (2013). MECP2 promotes cell proliferation by activating ERK1/2 and inhibiting p38 activity in human hepatocellular carcinoma HEPG2 cells. Cell Mol Biol.

[14] Tong D, Zhang J, Wang X, Li Q, Liu LY, Yang J, Guo B, Ni L, Zhao L, Huang C (2020). MeCP2 facilitates breast cancer growth via promoting ubiquitination-mediated P53 degradation by inhibiting RPL5/RPL11 transcription. Oncogenesis.

[15] Zhao L, Liu Y, Tong D, Qin Y, Yang J, Xue M, Du N, Liu L, Guo B, Hou N, Han J, Liu S, Liu N, Zhao X, Wang L, Chen Y, Huang C (2017). MeCP2 promotes gastric cancer progression through regulating FOXF1/Wnt5a/β-Catenin and MYOD1/Caspase-3 signaling pathways. EBioMedicine.

[16] Zhao LY, Tong DD, Xue M, Ma HL, Liu SY, Yang J, Liu YX, Guo B, Ni L, Liu LY, Qin YN, Wang LM, Zhao XG, Huang C (2017). MeCP2, a target of miR-638, facilitates gastric cancer cell proliferation through activation of the MEK1/2-ERK1/2 signaling pathway by upregulating GIT1. Oncogenesis.

[17] Qin Y, Zhao L, Wang X, Tong D, Hoover C, Wu F, Liu Y, Wang L, Liu L, Ni L, Song T, Huang C (2017). MeCP2 regulated glycogenes contribute to proliferation and apoptosis of gastric cancer cells. Glycobiology.

[18] Fan CC, Tsai ST, Lin CY, Chang LC, Yang JC, Chen GY, Sher YP, Wang SC, Hsiao M, Chang WC (2020). EFHD2 contributes to non-small cell lung cancer cisplatin resistance by the activation of NOX4-ROS-ABCC1 axis. Redox Biol.

[19] Shanmugasundaram K, Nayak BK, Friedrichs WE, Kaushik D, Rodriguez R, Block K (2017). NOX4 functions as a mitochondrial energetic sensor coupling cancer metabolic reprogramming to drug resistance. Nat Commun.

[20] El-Osta A, Kantharidis P, Zalcberg JR, Wolffe AP (2002). Precipitous release of methyl-CpG binding protein 2 and histone deacetylase 1 from the methylated human multidrug resistance gene (MDR1) on activation. Mol Cell Biol.

[21] Ma LC, Kuo CC, Liu JF, Chen LT, Chang JY (2008). Transcriptional repression of O6-methylguanine DNA methyltransferase gene rendering cells hypersensitive to N, N'-bis(2-chloroethyl)-N-nitrosurea in camptothecin-resistant cells. Mol Pharmacol.

[22] Zhu F, Wu Q, Ni Z, Lei C, Li T, Shi Y (2018). miR-19a/b and MeCP2 repress reciprocally to regulate multidrug resistance in gastric cancer cells. Int J Mol Med.

[23] Chahrour M, Jung SY, Shaw C, Zhou X, Wong ST, Qin J, Zoghbi HY (2008). MeCP2, a key contributor to neurological disease, activates and represses transcription. Science.

[24] Bedard K, Krause KH (2007). The NOX family of ROS-generating NADPH oxidases: physiology and pathophysiology. Physiol Rev.

[25] Gregg JL (2014). NADPH oxidase NOX4 supports renal tumorigenesis by promoting the expression and nuclear accumulation of HIF2alpha. Cancer Res.

[26] Boudreau HE, Casterline BW, Burke DJ, Leto TL (2014). Wild-type and mutant p53 differentially regulate NADPH oxidase 4 in TGF-b-mediated migration of human lung and breast epithelial cells. Br J Cancer.

[27] Crosas-Molist E (2017). The NADPH oxidase NOX4 represses epithelial to amoeboid transition and efficient tumour dissemination. Oncogene.

[28] Diaz B (2009). Tks5-dependent, nox-mediated generation of reactive oxygen species is necessary for invadopodia formation. Sci Signal..

