# Supplementary material for: MeCP2 confers 5-fluorouracil resistance in gastric cancer via upregulating the NOX4/PKM2 pathway
Source: Cancer Cell Int. 2022 Feb 18;22:86. doi: 10.1186/s12935-022-02489-y (PMC8857846; doi:10.1186/s12935-022-02489-y)
Supplement: Supplementary file 1 — Additional file 1. Sequences, antibodies, and patient characteristics in this study. [file 12935_2022_2489_MOESM1_ESM.doc]

**Table S1.** Sequences of siRNA

| Name | Sequence | |
| --- | --- | --- |
| negative siRNA (NC-siRNA) sense | 5′-UUCUCCGAACGUGUCACGUTT-3′ |  |
| negative siRNA (NC-siRNA) antisense | 5′- ACGUGACACGUUCGGAGAATT-3′ | |
| si-MeCP2-1 sense | 5′-GCUUAAGCAAAGGAAAUCUTT-3′ | |
| si-MeCP2-1 antisense | 5′-AGAUUUCCUUUGCUUAAGCTT-3′ | |
| si-MeCP2-2 sense | 5'-GCUUCCCGAUUAACUGAAATT-3' | |
| si-MeCP2-2 antisense | 5'-UUUCAGUUAAUCGGGAAGCTT-3' | |
| si-NOX4 sense | 5'-CCAUGUGCCGAACACUCUUTT-3' | |
| si-NOX4 antisense | 5'-AAGAGUGUUCGGCACAUGGTT-3' | |

**Table S2. Sequences of MECP2 MT Recombinant Plasmids**

| Name | Sequence |
| --- | --- |
| MECP2-WT MBD sequence | 5'-AGGGGCCCTATGTATGATGACCCTACACTGCCCGAGGGCTGGACCAGGAAACTGAAGCAGAGGAAGTCCGGA**AGGAGCGCCGGCAAATACGAT**GTCTACCTGATTAACCCCCAGGGCAAGGCCTTTAGATCCAAGGTGGAGCTG**ATCGCCTACTTTGAGAAGGTC**GGCGACACATCCCTAGACCCGAATGACTTCGACTTCACAGTGACCGGCAGAGGA-3' |
| MECP2-MT1 MBD sequence | 5'-AGGGGCCCTATGTATGATGACCCTACACTGCCCGAGGGCTGGACCAGGAAACTGAAGCAGAGGAAGTCCGGA**GATCAGTATCACGAAGGTCGC**GTCTACCTGATTAACCCCCAGGGCAAGGCCTTTAGATCCAAGGTGGAGCTGATCGCCTACTTTGAGAAGGTCGGCGACACATCCCTAGACCCGAATGACTTCGACTTCACAGTGACCGGCAGAGGA-3' |
| MECP2-MT2 MBD sequence | 5'-AGGGGCCCTATGTATGATGACCCTACACTGCCCGAGGGCTGGACCAGGAAACTGAAGCAGAGGAAGTCCGGAAGGAGCGCCGGCAAATACGATGTCTACCTGATTAACCCCCAGGGCAAGGCCTTTAGATCCAAGGTGGAGCTG**CATGACAAGCAGGCCGAGAGT**GGCGACACATCCCTAGACCCGAATGACTTCGACTTCACAGTGACCGGCAGAGGA-3' |

**Table S3. Primer Sequences Used for qRT-PCR** in This Study

| Gene | Sequence |
| --- | --- |
| MeCP2-F | 5'-GCCGAGAGCTATGGACAGCA-3' |
| MeCP2-R | 5'-CCAACCTCAGACAGGTTTCCAG-3' |
| NOX4-F | 5'-GGAGTTGACGTCGGAAT-3' |
| NOX4-R | 5'-AGTAACTTCGACTTTAAGGT-3' |
| β-Actin-F | 5'-TGGCACC CAGCACAATGAA-3' |
| β-Actin-R  ChIP-F  ChIP-R | 5'-CTAAGTCATAGTCCGCCTAGAAGCA-3'  5'-TAGTAGAGACAGGGGTTTCACAATC-3'  5'-ATAGAAATTGGTAGTTTGGGGCAGG-3' |

**Table S4.** Sequences of recombinant plasmids

| Name | Sequence |
| --- | --- |
| Negative control  MeCP2 shRNA | 5′-AAAAGAGGCTTGCACAGTGCATTCAAGACGTGCACTGTGCAAGCCTCTTTT-3′  5'-TGCTTAAGCAAAGGAAATCTCTCGAGAGATTTCCTTTGCTTAAGCTTTTTTC-3' |

**Table S5. Information on antibodies used for the correlation analysis**

| Antibody | WB | Specificity | | Company | |
| --- | --- | --- | --- | --- | --- |
| MeCP2 (sc-20700)  NOX4 (sc-518092)  PKM2 (ab137852)  β-Actin (sc-8432)  MeCP2 ChIP Grade (ab2828)  GFP ChIP Grade (ab290)  IgG ChIP Grade (ab171870) | 1:1000  1:1000  1:1000  1:3000  -  -  - | Rabbit polyclonal  Mouse Monoclonal  Rabbit polyclonal  Mouse Monoclonal  Rabbit polyclonal  Rabbit polyclonal  Rabbit polyclonal | Santa Cruz Biotechnology  Santa Cruz Biotechnology  Abcam  Santa Cruz Biotechnology  Abcam  Abcam  Abcam | |  |

**Table S6. Patient characteristics and clinicopathologic correlation of NOX4** expression

| Characteristics | Number of cases | | | NOX4 mRNA expression | | | P-value |
| --- | --- | --- | --- | --- | --- | --- | --- |
|  |  |  |  | High (n = 65) | | Low (n = 16) |  |
| Age | |  |  | |  | | 0.882 |
| ≥60 years | 38 | | 30 | | 8 | |  |
| <60 years | 43 | | 35 | | 8 | |  |
| Gender |  | |  | |  | | 0.675 |
| Male | 50 | | 39 | | 11 | |  |
| Female | 31 | | 26 | | 5 | |  |
| Histology |  | |  | |  | | 0.003* |
| Well | 39 | | 27 | | 12 | |  |
| Moderate | 20 | | 18 | | 2 | |  |
| poor | 22 | | 20 | | 2 | |  |
| Tumor size  <50 mm  ≥50 mm  Lymph node metastasis Yes  No  Lymphatic invasion  Yes  No  Venous invasion  Yes  No  T stage | 36  45  59  22  52  29  13  68 | | 23  42  52  13  42  23  9  56 | | 13  3  7  9  10  6  4  12 | | 0.001*    0.008*  0.871  0.106  0.013* |
| T1 | 29 | | 21 | | 8 | |  |
| T2 | 22 | | 16 | | 6 | |  |
| T3 | 18 | | 17 | | 1 | |  |
| T4 | 12 | | 11 | | 1 | |  |
| TNM Stage |  | |  | |  | | 0.198 |
| I | 20 | | 14 | | 6 | |  |
| II | 26 | | 21 | | 5 | |  |
| III | 20 | | 17 | | 3 | |  |
| IV | 15 | | 13 | | 2 | |  |

*P < 0.05
